# Cardiac rehabilitation and secondary prevention of CVD: time to think about cardiovascular health rather than rehabilitation

**DOI:** 10.1038/s44325-024-00017-7

**Published:** 2024-09-30

**Authors:** Julie Redfern, Robyn Gallagher, Andrew Maiorana, Dion Candelaria, Matthew Hollings, Sarah Gauci, Adrienne O’Neil, Georgia K. Chaseling, Ling Zhang, Emma E. Thomas, Gabriela L. M. Ghisi, Irene Gibson, Karice Hyun, Alexis Beatty, Tom Briffa, Rod S. Taylor, Ross Arena, Catriona Jennings, David Wood, Sherry L. Grace

**Affiliations:** 1 Institute for Evidence-Based Healthcare, Bond University, Gold Coast, NSW Australia; 2 Susan Wakil School of Nursing and Midwifery, Faculty of Medicine and Health, University of Sydney, Sydney, NSW Australia; 3 Curtin School of Allied Health, Curtin University, Bentley, WA Australia; 4 Allied Health Department, Fiona Stanley Hospital, Murdoch, WA Australia; 5 School of Health Sciences, Faculty of Medicine and Health, University of Sydney, Sydney, NSW Australia; 6 Institute for Mental and Physical Health and Clinical Translation, Deakin University, Geelong, VIC Australia; 7 Centre for Online Health, Centre for Health Services Research, The University of Queensland, Brisbane, QLD Australia; 8 Kite Research Institute, University Health Network, Toronto, Canada; 9 School of Medicine, University of Galway, Galway, Republic of Ireland; 10 Cardiology Department, Concord Hospital, ANZAC Research Institute, Concord, NSW Australia; 11 Alexis L Beatty, Departments of Epidemiology & Biostatistics and Medicine, University of California, California, USA; 12 University of Western Australia, Crawley, WA Australia; 13 School of Health and Well Being, College of Medical, Veterinary and Life Sciences, University of Glasgow, Glasgow, Scotland UK; 14 Department of Physical Therapy, College of Applied Health Sciences, University of Illinois Chicago, Chicago, IL USA; 15 National Institute for Prevention and Cardiovascular Health NIPC and University of Galway, Galway, Republic of Ireland; 16 Faculty of Health, York University, Toronto, ON Canada; 17 KITE & Peter Munk Cardiac Centre, University Health Network, University of Toronto, Toronto, ON Canada

**Keywords:** Cardiology, Health care

## Abstract

During the past century, there have been major developments in the medical and surgical treatment of cardiovascular disease (CVD). These advancements have resulted in more people surviving initial events and having reduced length of stay in hospital; consequently, there is an increasing number of people in need of ongoing and lifelong cardiovascular risk management. The physical and emotional effects of living with CVD are ongoing with broad challenges ranging from the individual to system level. However, post-discharge care of people with coronary disease continues to follow a 50-year-old cardiac rehabilitation model which focuses on the sub-acute phase and is of a finite in duration. The aim of this paper is to consider the concept of supporting survivors to live well with CVD rather than ‘rehabilitating’ them and propose factors for consideration in reframing secondary prevention towards optimizing cardiovascular health. We discuss deeply-held potential considerations and challenges associated with the concept of supporting survivors achieve optimal cardiovascular health and live well with CVD rather than ‘rehabilitating’ them. We propose the concept of 5 x P’s for reframing traditional cardiac rehabilitation towards the concept of cardiovascular health for survivors beyond ‘rehabilitation’. These include the need for personalization, processes, patient-centered care, parlance, and partnership. Taken together, consideration of challenges at the systems and population level will ultimately improve engagement with secondary prevention as well as outcomes for all people who need it.

## Introduction

Cardiovascular disease (CVD), including coronary heart disease (CHD) and stroke, relentlessly continues to be the greatest cause of mortality and disease burden across the globe^1^. Based on 2019 data, approximately one-third of global fatalities were attributable to CVD, which equates to almost 18 million deaths^1^. Importantly, among survivors of an acute coronary event, one in four experience at least one emergency hospital admission for CVD within 2 years^2^. Moreover, evidence recently showed that leading CVD risk factors significantly increased the risk of poor outcomes in those infected with coronavirus disease 2019 (COVID-19)^3^. In this context, the global CVD crisis that has persisted for decades has decreased human resiliency in the face of other health challenges, such as viral pandemics.

During the past century, there have been major developments in CVD management in terms of how a diagnosis is made, how arteries are revascularized, particularly in coronary vasculature, and the breadth and effectiveness of available medications for people with CVD^4^. These advancements have resulted in more people surviving initial events and having reduced length of stay in hospital^3^; consequently, there is an increasing number of people in need of ongoing and lifelong cardiovascular risk management^5^. As such, international groups and organizations have identified improved secondary prevention as an global priority^6,7^. In addition, the impact of receiving a diagnosis of CVD or surviving a heart attack is traumatic and life-changing^8^. The physical and emotional effects of living with CVD^9^ are ongoing with broad challenges ranging from the individual to system level^10^.

Global health systems are facing an escalating challenge. The combination of an aging population and decreased population resiliency due to unhealthy lifestyle behaviors coupled with medical advancements means more people are living longer with heart disease and its sequelae^11^. Increasing numbers of survivors are in need of ongoing care and support to make sustained change, minimize disability, and reduce risk of recurrent events. The concept of survivorship, where the focus is on promoting health and wellbeing beyond diagnosis and treatment, is well recognized in cancer care and research^12^. In contrast, post-discharge care of coronary disease continues to follow a 50-year-old cardiac rehabilitation model which focuses on the sub-acute phase, is finite in duration^5,13^ and lacks systems for improving the model based on consumer input^14^. The aim of this paper is to consider the concept of supporting survivors to live well with CVD rather than ‘rehabilitating’ them and propose factors for consideration in reframing secondary prevention towards optimizing cardiovascular health.

## Cardiac rehabilitation

Cardiac rehabilitation is a comprehensive secondary prevention model of care which is proven to mitigate the health and economic burden of CVD^15,16^. Cardiac rehabilitation programs deliver individualized, inter-professional care, including: clinical assessment, structured exercise training, patient and family education, cardiovascular risk factor management (e.g., smoking cessation advice where indicated and optimization of medications to control lipids and blood pressure) and psychosocial counseling^17^. The traditional model of cardiac rehabilitation is internationally accepted as comprising several sequential phases^17–19^. Phase 1 typically focuses on inpatient mobilization and introductory information but in recent years has been pared back to early discharge and changes in medical care. Phase 2 has traditionally been the primary focus – delivered by outpatient hospital-based programs run in groups attending for approximately six to 12 weeks^18^. Phase 3 is known as a maintenance phase of four to six months duration where people living with CVD continue their exercise and risk factor modification routine while returning to their regular life and work^20^. Programs vary somewhat in terms of dose and comprehensiveness^21^, likely impacted by several factors, including funding models within a given system^22^.

A large body of scientific research has highlighted the benefits of cardiac rehabilitation for those who attend^23–26^. These benefits include reduced risk of subsequent myocardial infarction, a modest reduction in all-cause mortality, and a considerable reduction in all-cause hospital admissions along with associated healthcare costs, increased functional/exercise capacity, and improved quality of life up to 12 months^23–26^. Evidence has highlighted the importance of comprehensive programs that manage multiple risk factors are most effective in terms of reducing all-cause mortality^26^.

Unfortunately, while major international guidelines strongly recommend cardiac rehabilitation for all with CVD^27–31^, research consistently shows that survivors have unacceptably poor rates of referral (30% of eligible)^32^, attendance (9% of eligible) and completion (<5% of all eligible)^33^. Further, the use of evidence-based medications and lifestyle improvements typically decline within six months^34,35^ of an acute event and are rarely sustained^36,37^. Contributing issues include that, historically consumers have not been involved in the reporting and design of cardiac rehabilitation programs. Health systems continue to be under-resourced leading to cardiac rehabilitation programs falling between the cracks of acute and primary care. Research in this area has also been under-funded and lacking national and international unity^38^. Global groups such as the International Council of Cardiovascular Prevention and Rehabilitation (ICCPR; www.globalcardiacrehab.com) and SOLVE-CHD (www.solvechd.org.au/) are collaborating to inform the conversation through unity and capacity building, but the challenge is complex and requires an interdisciplinary solution^38^.

## Understanding the historical context of cardiac rehabilitation

**Fig. 1:**
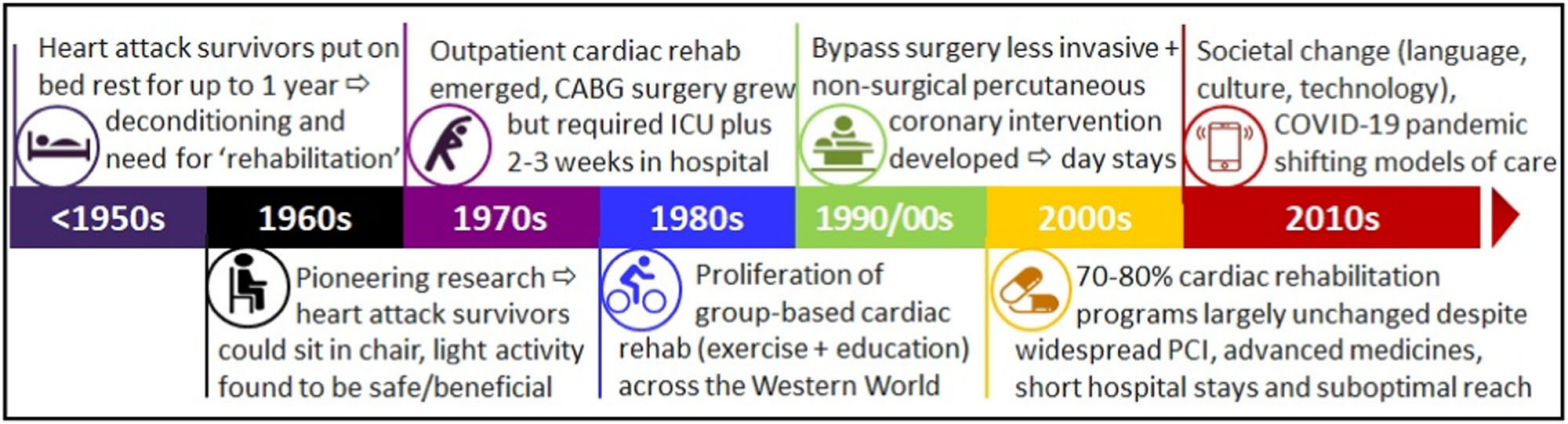
Historical context underscores the need for reform^5^. PCI, percutaneous coronary intervention.

Exploring the origins of cardiac rehabilitation provides insight into why current programs are structured as they are and why programs have been slow to adapt to the evolving needs of patients living with CVD and societal expectations^5^. These changes include significant shifts in culture, language, and diversity, substantial advancements in the medical and surgical management of CVD, and the rapid expansion of technology availability and capability (Fig. 1)^5^. Exploring the beginnings of cardiac rehabilitation informs our understanding of why current programs are designed as they are and how this has failed to adapt with changed needs of societies^5^.

Modern-day cardiac ‘rehabilitation’ was born at a time when bed rest and physical inactivity were recommended for people after a heart attack^39^. In the mid and late 20th century, survivors of heart attacks did require ‘rehabilitation’ - the timeline is detailed elsewhere but is represented schematically here in Fig. 1^5^. To summarize, despite being designed more than 60 years ago^40^, the traditional model of cardiac rehabilitation is still followed by 70-80% of programs globally in today’s vastly different societal and medical contexts^13,40^. That is, cardiac rehabilitation largely continues to follow a *’one size fits all*’ service-oriented model rather than a patient or survivor centered approach. Historically, programs have also been exercise-focussed with more contemporary literature highlighting the importance of comprehensive prevention with emphasis on behavioral approaches to address lifestyle as well as other pharmacotherapy for addressing clinical risk factors^26^. Taken together, programs lack flexibility and choice for people with CVD and hence their needs and preferences are not always paramount. Historical underpinnings are ultimately contributing to the sub-optimal referral, reach, participation and completion reflecting the need to reimagine cardiac rehabilitation service delivery in the 21st century.

## The numbers do not add up

Globalization (including migration) has resulted in economic development along with greater need to manage equity and diversity both within and between countries. For example, individuals who do not speak the language of the country in which they live, those who live in rural and remote geographical areas, those with socioeconomic disadvantage, people from culturally diverse backgrounds and women remain under-represented in cardiac rehabilitation^10^. International guidelines recommend all eligible people should be offered and participate in a cardiac rehabilitation program^27–31^. However, the numbers do not add up.

Based on existing resources, if all eligible people referred to traditional cardiac rehabilitation took up the opportunity to participate, health systems would not be able to meet the demand. For example, recent data from Europe suggests that cardiac rehabilitation programs lack around 3.5 million places annually^40^. In the United States of America, the Million Hearts initiative has set a national target of increasing cardiac rehabilitation participation from 20% to 70%^41^, which based on current case numbers would roughly represent an additional 1.7 million participants annually.

Although costs vary within and between countries, it is globally estimated a traditional program costs $US945 per person (calculated based on purchasing power parity)^22^. To meet the above stated targets, the increased participation in Europe (3.5 million participants x $US945) and the USA (1.7 million participants x $US945) would conservatively cost an estimated $US3.3 billion (~€3.02 billion) and $US1.6 billion (approximately €1.47 billion) respectively to implement. Although these costs are likely to be an underestimate in contemporary healthcare they nevertheless highlight the cost of delivering traditional cardiac rehabilitation to all who are eligible around the world. Despite documented cost benefits of cardiac rehabilitation in its traditional form^42^, it is unlikely international governments will have the funding to pay for such costs upfront despite hope of a return on investment in the following 5-10 years. Robust long-term research also lacking in this area.

When considered in this way, the upfront funding requirements, of traditional cardiac rehabilitation, for societies and governments are unrealistic within contemporary health budgets being highly unlikely to be in a position to deliver scale-up. Furthermore, these financial challenges are significantly greater in low and middle-income countries. Ultimately, it appears unfeasible to offer traditional group-based and in-person cardiac rehabilitation at scale to all people who are eligible. Financial constraints also highlight the need for modernization, greater flexibility, and potential expansion of innovative and virtual models for supporting CVD survivors in managing their life-long cardiovascular risk. Consideration of the concept of ‘proportionate universalism’ could be considered relevant where consideration of how systems could balance care among and between people who need it most also help address equity but also balance cost and availability^43,44^.

## Shifting the framing from ‘rehabilitation’ to ‘cardiovascular health’

The ‘cancer’ care and research community has embraced the importance of survivorship care with the aim of supporting people with cancer to participate fully in life in a meaningful way for the long term^45^. This need has arisen due to increasing cancer incidence rates (mainly resulting from an aging population and unhealthy lifestyle behaviors), earlier detection, and improved treatments and ongoing cardiovascular health^46^. Survivorship care in this area has a focus on strengths, a sense of vitality, living well and living a full life^47^. The same phenomena are occurring in relation to the number of people living with CVD. However, post-discharge care for people with CVD continues to focus on the above outlined time-limited ‘rehabilitation’ model^5,40^. Moreover, risk factors and secondary prevention recommendations to prevent recurrence are common to cancer and CVD^48,49^.

Survivorship care for people post-cancer has a focus on living well and promotes physical activity, a healthy diet and weight management, and recommended immunizations for survivors via care coordination that ensures all needs are addressed^45^. Associated strategies include appropriate screening, evaluation/assessment and treatment of contributing factors, education and counseling along with appropriate referrals^47^. These concepts are remarkably similar to those proposed in the Secondary Prevention for All in Need (SPAN) framework previously published in relation to CVD^50^. In SPAN, it is proposed that all people with CVD receive an assessment, education and personalized risk factor management along with follow-up with flexibility in format and duration^50^. At the same time, there are increasing calls for flexibility via virtual options for CVD prevention to achieve the preventive quadrella of referral, reach, participation and completion^5,7,10,51,52^. With this in mind, we propose the concept of lifelong cardiovascular health rather than a period of ‘rehabilitation’.

Some of the challenges for optimizing and maintaining cardiovascular health among people with CVD include the need for longer term support and care rather than a time-limited traditional rehabilitation program. This is embedded in the language and terminology used ubiquitously in the field, namely “rehabilitation”. This terminology itself is problematic because it conflicts with the concept of ongoing cardiovascular health or survivorship. However, current terminology is heavily embedded around the world including with international and country-specific cardiac rehabilitation organizations, clinician expectations and healthcare funders^52^. To enhance cardiovascular health, programs should strive to be more responsive to personal needs and preference to optimize patient-centredness of care^14,53^. Research has repeatedly found, in cardiology and other areas of health, that patient-reported outcomes and experience are inextricably linked with mortality rates, clinical effectiveness and safety^53–55^. More systematic collection of data, including patient-reported measures, would enable capitalization on opportunities through data science to facilitate improved care by enabling the identification of gaps, benchmarking and opportunities for better health.

## Five x P’s for consideration in reframing ‘cardiac rehabilitation’

Although, traditional cardiac rehabilitation remains beneficial for those who attend, improvements require greater continuity in care, flexibility, and consideration of financial feasibility at a population level. To work towards reframing, we suggest that consideration of the below five ‘P’s’ (1) personalization, (2) processes and systems, (3) patient-centered care, (4) parlance, and (5) partnership and unity (Box 1).

Box 1 Proposed 5 x P’s for reframing cardiac rehabilitation 1. Personalization

Consideration of approaches that embed personalization and flexibility into service delivery. The use of more personalized and digital support programs offers an opportunity to absorb some of the increasing diversity and patient load. For example, varied settings and models such as community, home-based approaches, and hybrid approaches with personalized education and management support based on the need and preference of people living with CVD. This includes consideration of multimorbidity where care is focused on the ‘whole’ person rather than the disease itself. In particular, digital strategies can offer light-touch solutions that have the potential to extend reach of programs^10^ and support for people with CVD in the longer term in the most cost-effective way^56^.

2. Processes and systems

Enhancing and implementing processes and systems for capturing and using data would enable a connected health system with built-in quality improvement systems. Current cardiac rehabilitation programs frequently lack the infrastructure and systems that support the collection and use of data for potential quality improvement, with many programs still using paper-based record-keeping^57^. Expanding access to streamlined and electronic data collection would offer an opportunity to leverage programs improvements and reach and ultimately ensure better care is offered to more people. Such data could subsequently be used to ensure ongoing preventive care is comprehensive and reaches all those who can potentially benefit. Data would help improve care for those who are eligible and participate but also identify those people who are falling through the ‘cracks’.

3. Patient-centered

Ensuring care is provided in a way that benefits people who need it in the best way to help them access it and achieve optimal outcomes. Flexibility service delivery along with collection of patient-reported measures with consumer involvement in program redesign would potentially facilitate these improvements. Improving patient-centered care and enhancing involvement and input would enable improved services in terms of quality and meaningfulness and for expanded programs beyond the traditional length of time to provide more lifelong cardiovascular health and support^14,50,53–55^.

4. Parlance

Parlance where the language and terminology used in the field of cardiac rehabilitation presents challenges. Progressing challenging conversations about terminology from ‘rehabilitation’ to the concept of cardiovascular health and life-long preventive care^5^. On balance, although a period of ‘rehabilitation’ is beneficial for some people with CVD, a more life-long and multifaceted prevention approach is needed for all if we are to reduce the CVD burden at the population level. As has been previously recommended this could include a universal definition and classification of preventive ‘rehabilitation’^52^.

5. Partnership and unity

Striving for stronger partnership and global unity with the common goal of optimizing cardiovascular health. Partnerships include consumers, stakeholders, policy-makers, clinicians, researchers to name a few. In terms of global unity, the ICCPR are working to understand and identify evidence-practice gaps from a global perspective with a focus on low-resource settings^40,58^. By bringing together the global cardiac rehabilitation community, more unity and sharing of challenges as well as collaboration on strategic directions can be achieved.

## Conclusion

In this paper, we share deeply held potential considerations and challenges associated with the concept of supporting survivors to achieve optimal cardiovascular health and live well with CVD rather than ‘rehabilitating’ them. We emphasize the importance of modernization and the escalating demands required to meet current and projected expanding needs within financial limits. Furthermore, highlighting the importance of contemporary models of cardiac rehabilitation are being developed to better align with other treatments, changing societies, and technological advancements. We propose the concept of 5 x P’s for reframing traditional cardiac rehabilitation towards the concept of cardiovascular health for survivors beyond ‘rehabilitation’. These include the need for personalization, processes, patient-centered care, parlance, and partnership. Taken together, consideration of challenges at the systems and population level will ultimately improve engagement with secondary prevention as well as outcomes for all people who need it.
